# A Preliminary Study on the Functional Benefits of Computerized Working Memory Training in Children With Pediatric Bipolar Disorder and Attention Deficit Hyperactivity Disorder

**DOI:** 10.3389/fpsyg.2019.03060

**Published:** 2020-02-05

**Authors:** Alessandra M. Passarotti, Livia Balaban, Liza D. Colman, Lindsay A. Katz, Nidhi Trivedi, Li Liu, Scott A. Langenecker

**Affiliations:** ^1^Department of Psychology, The University of Illinois at Chicago, Chicago, IL, United States; ^2^Institute for Health Research and Policy, The University of Illinois at Chicago, Chicago, IL, United States; ^3^Department of Psychology, Adler University, Chicago, IL, United States; ^4^Health Science Center, Texas Tech University, Lubbock, TX, United States; ^5^Department of Psychology, Roosevelt University, Chicago, IL, United States; ^6^The Chicago School of Professional Psychology, Chicago, IL, United States; ^7^School of Public Health, The University of Illinois at Chicago, Chicago, IL, United States; ^8^Department of Psychiatry, College of Medicine, University of Utah, Salt Lake City, UT, United States

**Keywords:** bipolar, ADHD, child, executive functions, attention, mood, cognitive training, working memory

## Abstract

Twenty-nine pediatric patients (age range, 10–16 years) with working memory (WM) deficits, including children with pediatric bipolar disorder (PBD) with and without attention-deficit hyperactivity disorder (ADHD) comorbidity and children with ADHD, underwent a Cogmed WM training program. For both patient groups, WM performance on Cogmed tasks and on the Digit Span test improved significantly after training. Moreover, the PBD group improved on Trails Making Test A and on the Inhibition Scale, the Behavior Regulation Index, and the Global Executive Composite of the Behavioral Rating Inventory of Executive Function. The ADHD group improved significantly on the Trails Making Test B, the Spatial Span Test, and the Reading Fluency Test of the Woodcock–Johnson III, as well as on depressive symptoms. The present findings suggest that working memory training is beneficial not only in youths with ADHD but also in youths with PBD. They also show evidence of near and far transfer of WM improvement in these patients, although in different ways for the two patient groups. Future studies examining the mechanisms of cognitive remediation in pediatric patients will aid in creating tailored illness-specific cognitive interventions.

## Introduction

The present study examined whether a computerized working memory (WM) training program may improve WM performance in youths with WM deficits. Our study included children and adolescents with pediatric bipolar disorder (PBD), with or without attention-deficit hyperactivity disorder (ADHD) comorbidity and children and adolescents with ADHD. Both groups of patients typically exhibit significant deficits in WM (Passarotti et al., 2016), which plays a key role in executive functions (Baddeley, 2003; D’esposito, 2007) as well as learning and academic skills, such as reading and math (Gathercole et al., 2016).

It is well-established that children with ADHD often exhibit WM and attention problems (Barkley, 1997; Rubia et al., 1999; Tamm et al., 2004;, Rich et al., 2006; Barkley, 2010; Passarotti et al., 2010a). Conversely, it has been acknowledged only recently that children with PBD exhibit not only chronic emotional dysregulation (Geller et al., 2002; Dickstein and Leibenluft, 2006; Galanter and Leibenluft, 2008) but also significant WM deficits (Pavuluri et al., 2009; Passarotti et al., 2010a; Singh et al., 2010a). Specifically, verbal WM impairment is the most consistent finding in PBD when examining different WM components (Joseph et al., 2008; Singh et al., 2010a).

To date, there is only partial behavioral differentiation of impulsivity, inattention, and disinhibition symptoms in PBD and ADHD (Geller et al., 1998; Galanter and Leibenluft, 2008; Pavuluri and Passarotti, 2008), which is in part due to high comorbidity rates (Singh et al., 2006), similar behavioral manifestations, and similar neural dysfunction in fronto-striatal systems in PBD (Rich et al., 2006; Leibenluft et al., 2007; Singh et al., 2010b) and in ADHD (Rubia et al., 1999; Tamm et al., 2004; Rich et al., 2006; Passarotti et al., 2010a). However, WM deficits are present in PBD regardless of comorbidity with ADHD (Pavuluri et al., 2006). Moreover, the WM deficits in PBD persist in the euthymic state and tend to worsen over time, even with good treatment outcome (Pavuluri et al., 2009). Therefore, the mechanisms underlying the WM deficits may differ in PBD and ADHD.

In terms of academic performance, WM deficits in PBD and ADHD result in an increasing gap in school performance relative to healthy peers, as schooling progresses and more complex skills need to be mastered. As a consequence, these children struggle to meet academic goals and experience increased frustration, hopelessness, and emotional dysregulation, especially in PBD. Moreover, while ADHD medications and interventions target cognitive symptoms, treatments for PBD focus primarily on mood symptoms and not on cognitive deficits. However, WM deficits lead to negative educational, occupational, and social outcomes both in PBD and ADHD youths (Rubia et al., 2001; Passarotti et al., 2007; Passarotti and Pavuluri, 2011). Therefore, personalized cognitive remediation may be a promising complementary approach to pharmacological treatments that may tap into the potential of adolescent brain plasticity to enhance cognitive functioning. If this type of cognitive intervention were to be successful, it could prevent worsening clinical outcome and years of functional loss in these chronic diseases.

Recent adult and child studies provide some initial indications of the effectiveness of cognitive remediation. A recent meta-analysis revealed that participants who completed the *Cogmed Working Memory Training^TM^ Program* (Cognitive Medical Systems AB, Pearson Assessments) improved on average 26% in visuospatial WM functions and 23% in verbal WM functions (Soderqvist and Bergman Nutley, 2015). A few child studies found transfer of performance improvement to non-trained WM tasks that were closely related, in terms of underlying cognitive processes, to the trained exercises, an effect called “near transfer” (Kronenberger et al., 2011; Van Der Donk et al., 2015). However, there are unclear results on whether trained skills can “generalize” to non-trained tasks that do not share similar cognitive processes with the trained tasks, which is called “far transfer” (Chacko et al., 2014; Simons et al., 2016). Evidence of far transfer after cognitive training would increase the functional and ecological validity of cognitive interventions.

Published studies on cognitive intervention in ADHD have yielded mixed results with regard to far transfer. Evidence of far transfer was found in a randomized controlled trial study with 7- to 12-year-old children with ADHD (Klingberg et al., 2005), where better WM performance after training generalized to performance on a span-board task, as well as to verbal WM, response inhibition, complex reasoning, and parental ratings of ADHD symptoms. In another study, children with poor WM skills improved their math performance after WM training (Holmes et al., 2009). Furthermore, Stevens et al. (2015) found that after Cogmed training adolescents with ADHD exhibited an increase in WM performance, a decrease in ADHD symptoms, and, notably, enhanced brain activity in frontal, temporal, and parietal regions that are part of WM circuits. However, Van Der Donk et al. (2015) did not find transfer of WM improvement to other academic domains in a randomized controlled trial study with a large sample of 8- to 12-year-old children with ADHD, which targeted academic outcome. Similarly, another RTC study by Chacko et al. (2014) found “near transfer” effects, with verbal and non-verbal WM improvement in children with ADHD after training, but did not find any evidence of “far transfer” to other non-trained functional domains. Lastly, a comprehensive meta-analysis that reviewed various cognitive training programs, including Cogmed, revealed improved performance on tasks similar to the trained ones (i.e., near transfer), but found inconsistent evidence on transfer of improvement to other domains (i.e., far transfer), such as executive functions, academic performance, non-verbal reasoning, and selective and sustained attention (Simons et al., 2016). Given the lack of clear-cut results, there is a strong a need for more developmental studies on cognitive intervention that would deepen our understanding of the cognitive mechanisms underlying “near” and “far” transfer and better define what type of cognitive training may foster “far transfer,” which has been so far elusive.

One electroencephalography study with adult BD found improvement in attentional brain function after an 8-week mindfulness/attention training intervention (Howells et al., 2012). Another very recent study with adult BD found improvement in daily-life WM and attention functions, as well as in verbal and visuospatial WM functions, after a computerized WM training (Lengvenyte et al., 2019). However, there are currently no published data on WM intervention in PBD, even though the potential benefits of this intervention on functional outcome in PBD has been well recognized (Dickstein et al., 2015). In spite of the existing literature on youths with ADHD, it is still an open question whether cognitive remediation is amenable also in youths with PBD because, in PBD, emotional dysregulation and cognitive deficits interact in unique ways that are markedly different from ADHD patterns (Rich et al., 2006; Galanter and Leibenluft, 2008; Passarotti et al., 2010a). Therefore, there is a need for a “proof of concept” study, investigating whether WM remediation may be useful in youths with PBD.

Most cognitive remediation studies with clinical population are adult studies. This study is the first to examine the effects of WM training concurrently in youths with PBD and youths with ADHD. This is particularly relevant because we need more developmental studies to clarify the effects of cognitive remediation in pediatric population. We also need more studies examining whether cognitive training may be a viable intervention in youths with mood dysregulation, such as PBD. Furthermore, because the phenotype of WM deficits in PBD and ADHD may differ, it is important to explore whether patterns of training-related WM improvement may differ between PBD and ADHD, and if so, what the underlying different mechanisms might be. Half of the PBD patients had a comorbid diagnosis of ADHD, which was not considered a reason for exclusion, given that many PBD patients have ADHD comorbidity (Rothman, 1990; Passarotti et al., 2007; Murthy et al., 2012; Passarotti et al., 2013). However, our analyses addressed potential effects of ADHD comorbidity on the training outcomes for the PBD patients.

The present study adopted the *Cogmed Working Memory Training^TM^ Program* (Cognitive Medical Systems AB, Pearson Assessments) and a battery of clinical scales and neuropsychological tasks to investigate training-related WM improvement and “near” and “far” transfer effects. Specifically, we wished to explore “far transfer” in terms of improvement of academic skills that rely heavily on WM, such as reading and math, and in terms of daily-life executive functions. Therefore, we employed the Math and Reading Fluency subtests of the Woodcock–Johnson III Tests of Achievements (Woodcock et al., 2001), the parental version of the Behavior Rating Inventory of Executive Function (BRIEF-PR) (Gioia et al., 2000), and outcome scales related to mood regulation and ADHD symptoms.

Our main study goal was to compare performance scores before and after the WM training for each patient group as well as between groups, for the Cogmed training tasks, the standardized clinical scales, and the neuropsychological WM tasks. This preliminary study did not include a healthy control (HC) group, rather just two patient groups, because our initial goal was to examine whether patients’ WM performance would improve after the cognitive training as compared to baseline. Therefore, each patient group served as its own “control” in terms of assessing training-related changes in WM performance. Moreover, the neuropsychological assessment scores were standardized, so that patients’ performance could be compared to standardized norms. However, future replications of this study would need to include a HC group to compare WM performance between HC and patients at baseline and also examine whether patient post-training performance may reach HC levels or not.

Based on the reviewed literature, we hypothesized that, after WM training, participants would show a significant improvement in Cogmed task performance as measured by the “Cogmed WM Index of Improvement,” calculated by a Cogmed algorithm (Klingberg et al., 2005; Stevens et al., 2015). We also expected that patients would show performance improvement on all or at least some of our WM tasks, especially those that shared similar WM processes with the Cogmed tasks. Specifically, our second goal was to examine whether there is “near transfer” and “far transfer” of improvement on Cogmed tasks to other non-trained tasks. We hypothesized that, for both groups, we would find more evidence for “near transfer” than for “far transfer” (Stevens et al., 2015; Simons et al., 2016). Our third goal was to examine potential group differences in the degree of improvement, depending on the measure. We hypothesized that ADHD and PBD may differ from each other in the degree of performance improvement depending on the task because of their different clinical phenotypes and most prominent symptoms, in spite of the discussed overlaps in WM deficits. Specifically, since patients with ADHD exhibit mainly WM and attention problems (Barkley, 1997; Rubia et al., 1999; Tamm et al., 2004; Rich et al., 2006; Passarotti et al., 2010a), we expected the ADHD group to show potentially greater improvements in these areas after the training. Since PBD has more prominent problems with behavior regulation, impulsivity, and emotion regulation (Geller et al., 2002; Dickstein and Leibenluft, 2006; Galanter and Leibenluft, 2008), we expected the PBD group to exhibit relatively more improvements in these domains.

Finally, since a key and understudied question on cognitive remediation is whether the training benefits may persist in time, our fourth, exploratory goal, was to examine whether improvements in performance might be retained in time (in full or partially) by testing patients again on the same non-trained tasks ∼4 months after the end of training.

## Materials and Methods

### Participants

Child and adolescent participants with a primary diagnosis of PBD or a primary diagnosis of ADHD were recruited from the Pediatric Mood Disorder Clinic, at the Colbeth Clinic, Department of Psychiatry, The University of Illinois at Chicago (UIC), and from the community in the Greater Chicago area. Participants exhibited significant WM deficits in school and daily functioning as reported by parents on a Cogmed WM questionnaire. Moreover, they exhibited clinically elevated *T* scores on the WM scale of the Behavioral Rating Inventory of Executive Function, Parental Report (BRIEF-PR) (Gioia et al., 2000), which is indicative of WM deficits. For all participants, consent from one parent or legal guardian and assent from the child participant were obtained. Participants were 10- to 16-year-old youths. The PBD patient sample (mean age = 12.5 ± 1.87 years) consisted of 16 pediatric patients with a diagnosis of PBD, type I or Type II, eight of which had a secondary diagnosis of comorbid ADHD, type combined. Thirteen children with ADHD, type combined, were also tested (mean age = 12.18 ± 1.78 years). Of the children with ADHD, three had a secondary diagnosis of depression, and two had a diagnosis of learning disability. None of the children with ADHD had a secondary diagnosis of PBD.

The cognitive intervention was proposed as complementary to pharmacological treatment and not as a substitute of it. For ethical reasons, if patients were medicated (including psychotropic medications and stimulants) at the time of enrollment into the study, we did not request any changes in medication regimen, to avoid worsening of symptoms. We accepted medications also at the time of testing to avoid differences in functioning between testing sessions and training sessions. Parents were asked to inform the researchers as soon as possible if there were any changes in the medication regimen while the child was enrolled in the study. Fifteen of the 16 PBD patients were already on a regimen of psychotropic medications when they started the Cogmed training. Nine of the 13 patients with ADHD were already on a medication regimen for ADHD symptoms at start of training.

Inclusion criteria were as follows: 8–19 years of age for all subjects; for the PBD group, axis I diagnosis of bipolar disorder type I or II, based on the Diagnostic and Statistical Manual of Mental Disorders IV-TR (DSM-IV-TR) (American Psychiatric Association, 2000); for the ADHD group, axis I diagnosis of ADHD type combined, based on DSM-IV-TR. For the PBD group, a diagnosis of comorbid ADHD based on the DSM-IV criteria was accepted because of the well-documented fact that many PBD patients have ADHD comorbidity (Rothman, 1990; Passarotti et al., 2007; Murthy et al., 2012; Passarotti et al., 2013). Patients were excluded from the study if they had a history of head trauma with loss of consciousness for more than 10 min, neurological symptoms, speech or hearing difficulties, pervasive developmental disorder, including autism, a primary diagnosis other than bipolar disorder or ADHD, and an IQ score lower than 70.

### Clinical and Demographical Assessment

The clinical diagnoses of PBD and ADHD were based on criteria from the DSM-IV-TR (American Psychiatric Association, 2000) because of the chronology of our study, since our data collection started while the DSM IV-TR was the current manual. The clinical diagnoses were formulated by clinicians at the UIC Pediatric Mood Disorder Clinic. In addition, for research purposes, several clinical scales were administered to all participants by trained research assistants in our Developmental Cognitive Neuroscience Laboratory: the Kiddie Schedule for Affective Disorders and Schizophrenia—Present and Lifetime version (Kaufman et al., 1997; Geller et al., 1998), supplemented by the mood disorders module from the Washington University in St. Louis Kiddie Schedule for Affective Disorders and Schizophrenia (Kaufman et al., 1997; Geller et al., 1998); the Young Mania Rating Scale (YMRS) (Young et al., 1978) which assessed mania symptoms; the Child Depression Rating Scale-Revised (CDRS-R) (Poznanski et al., 1979), which assessed depression symptoms; the Conners’ Parent Rating Scale-Revised (CPRS-R) (Conners et al., 1998), which assessed ADHD symptoms. Patient groups were matched based on age, gender, and IQ. For every participant, IQ was estimated using the Wechsler Abbreviated Scale of Intelligence (WASI) (Wechsler, 1999).

The study was approved by The University of Illinois at Chicago (UIC) Institutional Review Board (IRB).

### The Working Memory Intervention

All participants were assessed at two main time points at our laboratory: before and after the Cogmed training (i.e., within 2 weeks from the end of training, based on the family availability). Moreover, for participants who were available to return to the laboratory, there was a follow-up testing session at ∼4 months from the end of training.

For our study, we adopted the *Cogmed Working Memory Training^TM^ Program* (Cognitive Medical Systems AB, Pearson Assessments), *RM version*, which was specifically designed for school-age children. Our overarching rationale for the WM training is that it must start by practicing with basic WM processes (such as rehearsal of numbers to be remembered) to increase WM capacity and processing efficiency, which in turn may foster better higher-order cognitive processes (i.e., far transfer) (Klingberg et al., 2004, [Bibr B26][Bibr B24]). To this goal, we chose the Cogmed Working Memory Training Program, which is particularly suited for pediatric and clinical population because it is “adaptive,” in that it uses a training algorithm that adjusts the task difficulty level on a trial-by-trial basis depending on the individual’s performance (Klingberg et al., 2004). Another advantage of this program is that it relies on implicit learning, and not complex explicit strategies, to strengthen WM functions and capacity. The implicit learning approach is more suitable for children with PBD or ADHD, who are already dealing with significant mood dysregulation, poor ability to focus, and high frustration.

Participants were assessed approximately 1–2 weeks before the training, then again within ∼2 weeks from end of training, and at a 4-month follow-up. A certified Cogmed coach (AMP) trained the research staff and supervised the participants’ training. AMP met with the child and family before and after the Cogmed training and at the 4-month follow-up. Parents supervised the children during training at home and were encouraged to provide meaningful rewards at the end of each session and then a greater reward at the end of the training. The training consisted of 25 “self-paced” sessions, carried out between three and five times per week, which lasted on average 35–45 min. Seven Cogmed exercises were presented on each training session (i.e., Visual Data Link, Rotating Data Link, Data Room, Input Module with and without Lid, Stabilizer, and Rotating Dots), and five exercises were presented for a portion of the sessions (i.e., Decoder, Asteroids, Sorter, 3D Cube, Space Whack). Supplementary Table S1 provides a detailed description of each Cogmed task. In terms of the type of WM processes engaged by the training, nine of the training tasks utilize visuospatial stimuli and engage primarily visuospatial WM. Three of the training tasks (i.e., input module and input module with lid, decoder) utilize verbal stimuli and engage primarily verbal WM.

### Neuropsychological Assessment

All participants were assessed before and after the cognitive training. Moreover, for participants who were available to come back, there was a 4-month follow-up session. We adopted the following neuropsychological battery*:* (a) *Estimated IQ*, at baseline, two subtests from the WASI (Wechsler, 1999), namely, Vocabulary and Matrix Reasoning, were used to estimate global intellectual functioning and derive the Full-Scale IQ; (b) *Attention and Working Memory* (including attention, WM, and processing speed), Trail Making Test (TMT) A (Reitan, 1958), Digit Span Test Forward (WISC III) (Wechsler, 1991); Spatial Span Task—Forward (Wechsler Non-verbal Scale of Ability, Spatial Span test) (Massa and Rivera, 2009); (c) *Executive Functions* (including WM, cognitive flexibility, and processing speed), TMT B (Reitan, 1958); Digit Span Test Backward (WISC III) (Wechsler, 1991); Spatial Span Task Backward (Wechsler Non-verbal Scale of Ability, Spatial Span test) (Massa and Rivera, 2009); and (d) *Academic Skills*, Reading Fluency Test (assessing reading comprehension skills) and Math Fluency Test (assessing math and calculation skills), and Woodcock–Johnson Tests of Achievement (Woodcock et al., 2001).

Moreover, the BRIEF-PR (Gioia et al., 2000) was administered to assess executive function in daily life. The BRIEF*-*PR is a 86-item scale (items rated as “never,” “sometimes,” or “often”) for parental report on child’s behaviors, consisting of eight clinical scales measuring different aspects of executive functioning: Inhibition, Shift, Emotional Control, Initiation, Working Memory, Plan/Organize, Organization of Materials, and Monitor. The eight scales compose a Behavioral Regulation index (Inhibition, Shift, and Emotional Control) and a Metacognition index (Working Memory, Plan/Organize, Monitor, and Organization of Materials), which in turn compose a Global Executive Composite (GEC). Note that for this scale, higher T scores indicate greater impairment. Scores that are above 1 SD, or 10 points above the mean of *T* = 50, are considered clinically significant.

#### Stop Signal Task (SST)

We adopted a pediatric version of the SST created in our laboratory (Passarotti et al., 2010b). The SST measures the ability to inhibit prepotent motor responses, as well as WM, attention, and executive functions (Senderecka et al., 2012). On “go” trials participants pressed either a right or left key in response to a green circle appearing on either the right or left side of the computer screen. On “stop” trials, a red circle (i.e., stop sign) appeared at the center of the screen cueing participants to inhibit their motor response. The stop sign appeared randomly between 0 and 270 ms following the onset of the “go” sign. Sixty percent of the trials were go trials, and 40% were stop trials. Go trials were represented at the end of the task when a participant’s reaction time (RT) on go trials was too slow (i.e., RT > 650 ms). RT and accuracy were recorded for go trials, and accuracy was recorded for stop trials. For the purposes of this study, we focused our analyses on stop and go trial accuracy, to examine the ability to engage inhibition and sustained attention processes, respectively.

### Examining Near and Far Transfer

We conceptualized post-training improvements in TMT A, Digit Span, and Spatial Span tasks as evidence of “near transfer” because these tasks share basic cognitive processes with the trained Cogmed tasks. We conceptualized post-training improvements in TMT B, in the Math and Reading Fluency tests from the Woodcock–Johnson III, and the Stop Signal Task, as evidence of “far transfer” since these measures do not share basic processes with the trained Cogmed tasks. Similarly, we considered any post-training improvement in mood symptoms, as measured by the YMRS and CDRS-R scales or in ADHD symptoms, as measured by the CPRS-R scale, as evidence of “far transfer”.

### Statistical Data Analyses

#### Categorical Variables Analyses

Fisher’s exact tests were carried out for categorical variables (i.e., gender, race). Statistical Package for Social Sciences 24 (SPSS 24) was used to conduct *t* tests on demographic data (at baseline only), tasks, and clinical measures (at baseline, post-training, and follow-up). Paired *t* tests were employed to compare baseline and post-training task performance within group, while independent *t* tests were used for between-groups comparisons on the tasks.

#### Examining Within Group and Between Group Differences in Performance Before and After the Cogmed Training

Our primary analyses compared patients’ performance data at two main time points: before training (baseline) and after training. Univariate ANOVAs were also conducted for all tasks and scales to examine whether ADHD comorbidity in PBD, as a covariate, may have affected baseline performance or improvement in PBD, as well as group differences in improvement.

#### Examining Training Effects as Assessed by the Cogmed WM Improvement Index

To assess WM training effects, a Cogmed algorithm was used to calculate a “WM Improvement Index,” resulting from subtracting the *mean training level* on the third day (which is used as a baseline score to allow for performance stabilization) from the mean training level for the child’s best performance in the last 5 days of the training. Mean training level is the mean span length of letters, numbers or locations, depending on the exercise, that the child can remember on a certain day.

#### Examining Group Differences in the Extent of Improvement After Cogmed Training

We carried out *t* tests to compare the PBD and ADHD groups on improvement scores for each measure. The “improvement score” was derived by subtracting pretraining scores from post-training scores for each measure. Univariate ANOVAs examined effects of ADHD comorbidity in PBD with regard to improvement.

#### Examining Differences in Performance at a 4-Month Follow-Up Compared to End of Training

We conducted exploratory analyses to compare performance scores at a 4-month follow-up with those at the end of training to examine whether the training benefits may be retained in time.

#### Neuropsychological Assessment

For TMT A and B, *Z* scores were calculated based on raw scores from completion time (where higher *Z* scores correspond to higher completion times). For the Digits Span Test, raw data were transformed into scaled scores (ss: mean = 10, SD = 3), and a composite scaled score was obtained after summing the Forward and Backward digit span scores. For the Spatial Span Test, we calculated forward and backward raw scores, as well as scaled scores for the Spatial Span total score. For the two tests, forward and backward digit span scores were also examined separately. Specifically, comparisons of longest digit span forward (LDSF), longest digit span backward (LDSB), longest spatial span forward, and longest spatial span backward were carried out before and after training. For the Woodcock–Johnson tests, standardized scores were calculated (SS: mean = 100, SD = 15). For the BRIEF-PR and CPRS-R scales, the raw scores for each subscale were transformed into *T* scores (with mean = 50 and SD = 10).

#### Computerized SST

For the SST, we calculated mean accuracy for go and stop trials and compared them within and between group before and after training.

#### Correlation Analyses

Exploratory correlations analyses were carried out to explore potential correlations between Cogmed WM Improvement Index and scores at baseline measures of interest.

## Results

We summarize below the main results for *t* tests on group performance related to our tasks or scales and results from univariate ANOVAs on the effects of ADHD comorbidity in PBD.

Table 1 shows demographic data, WASI Full Scale IQ scores, Cogmed WM Start Index, Cogmed WM Max Index, and Cogmed WM Improvement Index for the PBD and ADHD groups.

**TABLE 1:** Demographic characteristics, WASI-FSIQ scores and Cogmed working memory index scores for patients with PBD and patients with ADHD.

	**PBD Mean (*SD*)**	**ADHD Mean *(SD)***	**Group Difference Statistics**
Age (in years)	12.50 *(1.87)*	12.18 *(1.78)*	*p* > 0.05
Gender			Fisher’s *p* > 0.05
*Male*	9	9	
*Female*	7	4	
**Race/Ethnicity**			Fisher’s *p* >0.05
*Caucasian*	11	9	
*Asian*	3	0	
*African-American*	1	3	
*Hispanic*	0	1	
*Unanswered*	1	0	
WASI-FSIQ	101.42 *(15.44)*	106.44 *(15.53)*	*p* > 0.05
CogWM Start Index	71.44 *(19.58)*	74.50 *(15.51)*	*p* > 0.05
CogWM Max Index	94.38 (16.64)**	106.50 (25.26)**	*p* > 0.05
CogWM Improvement Index	22.94 *(6.78)*	32 *(21.04)*	*p* > 0.05

FSIQ = Full scale intelligence quotient. * = Significant within-group difference in performance from baseline to after training at *p* < 0.05. ** = Significant within-group difference in performance from baseline to after training at *p* < 0.01.

### Demographic Results

No significant group differences were found with regard to demographics and IQ scores (all *P* < 0.05).

### Cogmed WM Start Index and Cogmed WM Improvement Index Results

As shown in Table 1, the PBD and ADHD groups did not differ in Cogmed WM Start Index at the beginning of training, suggesting comparable WM deficits at baseline. Importantly, each group showed a significant improvement in performance after training as measured by the Cogmed WM Improvement Index (i.e., the difference between the Cogmed WM Start Index and the Cogmed WM Max Index). However, there were no significant group differences in improvement. In fact, while ADHD had a slightly higher Cogmed WM Improvement Index than PBD after training (medium effect size, Cohen’s *d* = 0.58), the difference was not significant (*P* > 0.05).

A univariate ANOVA with ADHD comorbidity as covariate revealed no significant effects of ADHD comorbidity on the results (all *P* > 0.05).

### Neuropsychological Assessment Results

Table 2 illustrates results for the neuropsychological tests in PBD and ADHD. Below, we briefly report the main results.

**TABLE 2:** Pre- and post-training neuropsychological test scores for patients with PBD and patients with ADHD.

	**PBD**		**ADHD**	
	**Pre-Training Mean (*SD*)**	**Post-Training Mean (*SD*)**	**Pre-Training Mean (*SD*)**	**Post-Training Mean (*SD*)**
WJ-III Reading Fluency Standard Score	104.00 (*19.12*)^■^	102.50 (*19.20*)	86.00 (*10.95*)^■^	95.70 (*14.14*)*
WJ-III Math Fluency Standard Score	87.29 (*14.87*)	88.43 (*15.15*)	80.50 (*15.59*)	83.00 (*14.46*)
TMT A Z-score	−4.35 (*3.44*)	−2.53 (*2.26*)**	−5.33 (*4.70*)	−3.00 (*2.50*)
TMT B Z-score	−6.55 (*5.26*)	−5.77 (*5.13*)	−4.29 (*2.43*)	−4.09 (*3.52*)**
Digit Span Task Scaled Score	8.86 (*3.11*)	11.50 (*3.90*)**	8.00 (*2.13*)	10.12 (*2.95*)*
*Longest Digit Span Forward (LDSF)*	6.36 (*1.34*)	6.57 (*1.60*)	5.67 (*0.65*)	6.75 (*1.22*)*
*Longest Digit Span Backward (LDSB)*	3.71 (*1.14*)	5.00 (*1.47*) **	3.42 (*0.67*)	3.83 (*0.58*)
Spatial Span Task T-Score	47.07 (*10.98*)	51.36 (*8.93*)	45.50 (*7.26*)	55.92 (*6.07*)**
*Longest Spatial Span Forward (LSSF)*	5.14 (*1.23*)	5.57 (*1.02*)	4.67 (*0.89*)	6.12 (*1.11*)**
*Longest Spatial Span Backward (LSSB)*	4.54 (*1.05)*	5.08 (*1.04*)	4.59 (*0.90*)	5.08 (*0.79*)

* = Significant within-group difference in performance from pre- to post-training at *p* < 0.05. ** = Significant within-group difference in performance from pre- to post-training at *p* < 0.01. ^■^ = Significant between-group difference in performance from pre- to post-training at p < 0.05. Note: The black symbol ^■^ in each group’s column (at pre- or post-training) indicates a significant between-group difference at *p* < 0.05.

#### Woodcock–Johnson III Reading and Math Fluency Tests

The PBD group had a significantly higher score than the ADHD group on the Reading Fluency Test before the training. The PBD group did not show a significant improvement on this test after training. By contrast, the ADHD group showed a significant improvement on this test and did not differ significantly from PBD anymore after the training. No significant results in either group were obtained for the Math Fluency Test. A univariate ANOVA with ADHD comorbidity as covariate revealed no significant effects of comorbidity on PBD results, before or after the training (all *P*s > 0.05).

#### TMT A and TMT B

Regarding TMT A, the two groups did not differ significantly at baseline or after training. The PBD group demonstrated significant improvement on TMT A after training, while the ADHD group just missed a significant level of improvement on this test (*P* = 0.06) (medium effect size, Cohen’s *d* = 0.73). Regarding TMT B, only the ADHD group showed a significant improvement after training. The PBD and ADHD groups did not differ on TMT B scores before or after the training.

Univariate ANOVAs did not show significant effects of ADHD comorbidity for TMT A results. However, for TMT B, there was a significant effect of ADHD comorbidity on PBD performance before training [*F*(1,28) = 5.07, *P* = 0.03], in that the PBD group had higher (i.e., worse) scores than the ADHD and the Comorbid group before training. There were no other significant group differences or effects of comorbidity after training.

#### Digit Span Test

**FIGURE 1:**
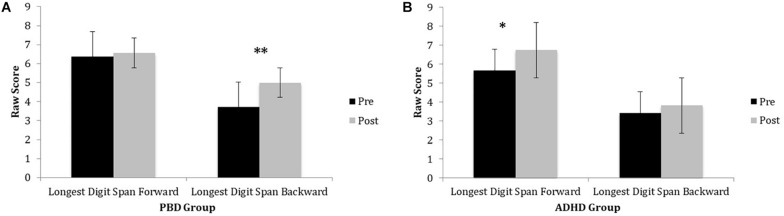
Digits Span Task performance in PBD and ADHD before and after Cogmed training. **(A)** Bar graph representing raw scores for the PBD group. **(B)** Bar graph representing raw scores for the ADHD group. PBD, pediatric bipolar disorder; ADHD, attention deficit-hyperactivity disorder; Pre, pre-training scores; Post, post-training scores. ^∗^*p* < 0.05; ^∗∗^*p* < 0.01.

Both the PBD and the ADHD group exhibited significant improvement on the Digit Span Test after training. However, they did not differ significantly from each other, at either time point. Figure 1 illustrates raw LDSF and LDSB scores on the Digit Span Test before and after training for PBD (Figure 1A) and ADHD (Figure 1B). The PBD group showed a significant improvement only for the LDSB but not for the LDSF. The opposite pattern was true for the ADHD group, which showed significant improvement only for the LDSF, but not for the LDSB (medium effect size; Cohen’s *d* = 0.65). The two groups did not differ significantly on LDSF or LDSB scores after training. For LDSB, the PBD group exhibited higher scores than the ADHD group at baseline (large effect size; Cohen’s *d* = 1.05).

Univariate ANOVAs did not show significant effects of ADHD comorbidity on PBD results before or after the training (all *P* > 0.05).

#### Spatial Span Test

**FIGURE 2:**
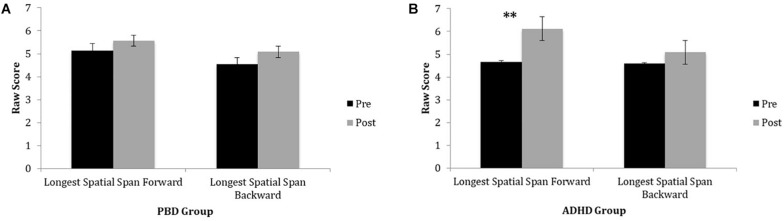
Spatial Span Task performance in PBD and ADHD before and after Cogmed training. **(A)** Bar graph representing raw scores for the PBD group. **(B)** Bar graph representing raw scores for the ADHD group. PBD, pediatric bipolar disorder; ADHD, attention deficit-hyperactivity disorder; Pre, pre-training scores; Post, post-training scores. ^∗∗^*p* < 0.01.

Figure 2 illustrates raw LDSF and LDSB scores on the Spatial Span Test before and after training for PBD (Figure 2A) and ADHD (Figure 2B). Only the ADHD group exhibited a significant improvement on the Spatial Span Test after training. For this test, the ADHD group also showed a significant improvement on the longest spatial span forward after training. For the longest spatial span backward, there were no significant improvements after training in either group (medium effect size; PBD: Cohen’s *d* = 0.52; ADHD: Cohen’s *d* = 0.58) (Figures 2A,B). Univariate ANOVAs did not show any significant effects of ADHD comorbidity before or after the training (all *P* > 0.05).

### Clinical Questionnaires

Clinical scale results are illustrated in Table 3.

**TABLE 3:** Pre- and post-training clinical scale scores for patients with PBD and patients with ADHD.

	**PBD**		**ADHD**	
	**Pre-Training Mean (*SD*)**	**Post-Training Mean (*SD*)**	**Pre-Training Mean (*SD*)**	**Post-Training Mean (*SD*)**
YMRS	15.56*(10.12)*^■^^■^	12.64 *(8.61)*^■^	4.8*(2.89)*^■^^■^	4.4*(2.62)*^n^
CDRS-R	29.00*(9.29)*	27.14*(5.82)*^■^	23.50 *(4.74)*	21.50 *(3.87)^∗^*^■^^■^
CPRS-RTotal ADHD Index	74.38*(8.04)*	73.00 *(10.25)*	71.08*(13.81)*	66.36*(9.16)*
*Oppositional*	66.69^■^*(11.75)*	65.14 *(9.23)*^■^	55.25^■^*(12.14)*	54.72*(11.59)*^■^
*Inattentive*	71.69*(13.96)*	70.00*(9.98)*	71.00*(15.44)*	64.18*(11.49)*
*Hyperactive*	74.13*(11.10)*	69.14 *(13.11)*	63.00^■^*(13.42)*	60.55*(12.40)*^■^
**BRIEF-PR**				
*Inhibition*	72.07 *(11.95)*^■^	64.00*(12.17)*^∗∗^	58.30 *(13.38)*^■^	59.80 *(15.27)*
*Shifting*	68.43 *(10.50)*^■^^■^	64.21 *(10.19)*	53.30 *(10.62)*^■^^■^	54.90 *(12.49)*
*Emotional Control*	71.50 *(11.93)*^■^	66.57 *(10.80)*^■^^■^	49.50 *(13.54)*^■^^■^	48.80 *(8.29)*^■^
*Initiation*	66.71 *(7.23)*	64.50 *(11.18)*	58.50 *(13.68)*	58.10 *(9.89)*
*Working Memory*	70.43 *(9.14)*	67.07 *(9.64)*	71.40 *(10.28)*	70.50 *(11.60)*
*Planning/Organization*	68.71 *(10.0)*	69.29 *(11.82)*	67.10 *(12.48)*	62.70 *(12.28)*
*Organization of Materials*	62.07 *(10.66)*	62.29 *(14.36)*	59.40 *(8.71)*	59.80 *(11.14)*
*Monitoring*	75.14 *(5.56)*^■^^■^	71.14 *(11.72)*	65.70 *(11.17)*^■^^■^	64.70 *(9.10)*
*Behavior Regulation Index (BRI)*	75.07 *(9.69)*^■^	68.14 (11.67)*	53.70 *(10.12)*^■^	54.50 *(9.63)*
*Metacognition Index (MI)*	73.43 *(9.85)*	73.57 *(13.15)*	67.60 *(11.67)*	68.70 *(11.53)*
*Global Executive Composite (GEC)*	74.29 *(7.30)*^■^^■^	68.86 *(9.12)*^∗^	63.80 *(11.22)*^■^^■^	62.00 *(9.43)*

* = Significant within-group difference in performance from pre- to post-training at *p <* 0.05. ** = Significant within-group difference in performance from pre- to posttraining at *p <* 0.01. ^■^ = Significant between-group difference in performance from pre- to post-training at *p <* 0.05. ^■■^ = Significant between-group difference in performance from pre- to post-training at *p <* 0.01. Note: The black symbols ^■^ or ^■■^ in each group’s column (at pre- or post-training) indicate a significant between-group difference at *p <* 0.05 or *p <* 0.01, respectively.

#### YMRS

As expected, the PBD group had significantly higher YMRS scores than the ADHD group both before and after the training. The PBD group exhibited no significant improvement in YMRS scores after the training. A univariate ANOVA showed no significant effect of ADHD comorbidity on PBD results before or after the training (all *P* > 0.05).

#### CDRS-R

Before the training, the ADHD and PBD groups did not differ in severity of depression symptoms, as measured by the CDRS-R. However, they differed significantly after training. In fact, only the ADHD group showed a significant reduction in depression symptoms after training, with post-training scores significantly lower than those for PBD. A univariate ANOVA revealed no significant effects of ADHD comorbidity on PBD results before or after the training (all *P* > 0.05).

#### CPRS-R

There were no group differences on the total ADHD index score before or after training. Neither group improved significantly on this measure after training. Moreover, while before training the PBD group had significantly worse scores than the ADHD group on the Oppositional scale of the CPRS-R, after training, the PBD group had significantly worse scores than the ADHD group on both the Oppositional and Hyperactive scales. No other results were significant.

Notably, a univariate ANOVA found a significant effect of ADHD comorbidity on post-training scores in PBD, for the Hyperactive [*F*(1,25) = 5.78, *P* = 0.03], and the Oppositional scale [*F*(1,25) = 4.24, *P* = 0.05]. The PBD-only group improved significantly less than the Comorbid group for these two subscales. These results suggest that in terms of selective ADHD symptoms, PBD patients with ADHD comorbidity benefited more from training than patients with only a diagnosis of PBD.

#### BRIEF-PR Scale

Table 3 illustrates mean *T* scores on the BRIEF-PR scale for the PBD and ADHD groups, before and after training.

While before training, the PBD group had clinically elevated scores on all the BRIEF subscales (i.e., *T* > 60), after training, the PBD group showed an improvement in Inhibition, BRI, and GEC scores. Before training, the ADHD group had clinically significant deficits on Working Memory, Planning/Organization, Monitoring, GEC, and Metacognition index scores. After training, ADHD did not show any significant improvement on this scale.

Another important result is that, before training, PBD had more clinically elevated scores than ADHD on several scales, including Inhibition, Shifting, Emotional Control, Monitoring, and the BRI and GEC. However, after training, PBD had higher scores than ADHD only for the Emotional Control scale. A univariate ANOVA found a significant effect of ADHD comorbidity on PBD scores only for the BRIEF-PR WM scale before the training [*F*(1,28) = 9.03, *P* = 0.006], in that before the training, the Comorbid group (*T* = 77) had higher scores (i.e., worse symptoms) than the PBD group (*T* = 65). This effect was not significant anymore after the training (PBD, *T* = 64; Comorbid, *T* = 71), suggesting beneficial effects of WM training especially in the Comorbid group.

### Computerized Inhibition Task: The SST

When comparing the PBD and ADHD groups on accuracy scores for the SST go and stop trials before the training, no significant group differences were found (all *P* < 0.05). The average go and stop trial accuracy for PBD were 68% (SD = 0.19) and 55% (SD = 0.16), respectively; for ADHD, they were 68% (SD = 0.17) and 57% (SD = 0.09), respectively.

Moreover, there were no significant group differences in SST go and stop trials accuracy after the training (*P* < 0.05). After training, the average go and stop trial accuracy for PBD were 73% (SD = 0.19) and 56% (SD = 0.15), respectively; for ADHD, they were 74% (SD = 0.14) and 58% (SD = 0.11), respectively.

A univariate ANOVA showed no significant effects of ADHD comorbidity (*P* > 0.05).

### Analysis of Group Differences in the Extent of Improvement After Cogmed Training

*t* test results indicate significant group differences in the degree of post-training improvement with regard to two scales of the BRIEF-PR, specifically BRI [*t*(24) = −2.21, *P* = 0.04] and Inhibition [*t*(23) = −3.31, *P* = 0.003], where PBD improved to a greater extent than ADHD. Also for the Spatial Span Task, there was a significant group difference, in that ADHD improved to a greater extent than PBD [*t*(23) = −2.20, *P* = 0.04] after training. For all other measures, the degree of improvement did not differ significantly for ADHD and PBD. Univariate ANOVAs revealed no significant effects of ADHD comorbidity for the two significant BRIEF-PR measures.

### Exploratory Analyses: Examining Differences in Performance at a 4-Month Follow-Up Compared to End of Training

Only a small subset (*N* = 11) of the original sample returned to the laboratory after 4 months for further testing. Therefore, we were able to conduct only exploratory analyses on a small sample to compare 4-month follow-up scores with scores at the end of training. For this analysis, we collapsed our data across diagnosis. The sample included four patients with ADHD and seven patients with PBD (four of which had ADHD comorbidity). The results indicate that overall at 4 months, the patients’ scores did not differ significantly from their scores at the end of training, with the exception of the Digit Span Task, for which the 4 months follow-up scores actually improved. Specifically, no significant differences were found for the following clinical scales: YMRS (*P* = 0.96), CDRS (*P* = 0.28), CPRS-R (all *P* > 0.05), and BRIEF-PR (all *P* > 0.05). Regarding the tasks, there were no significant differences for TMT A and B (*P* = 0.69; *P* = 0.35, respectively) or for the Spatial Span Test (*P* = 0.82). However, for the Digit Span Test, which measures verbal WM functions, we found a significant difference [*t*(10) = 7.64, *P* = 0.0001] in that scores improved after 4 months (SS = 15.73) compared to the end of training (SS = 11.10). We were not able to obtain sufficient follow-up data for analyses for the Woodcock–Johnson Math and Reading tests and for the SST because of technical difficulties.

In sum, this pattern of results, while very preliminary, suggests a potential for retention of the training benefits in time.

### Exploratory Correlation Analyses

Finally, to further explore whether number of sessions completed, baseline severity of symptoms or baseline WM performance had an effect on the CogWM Improvement Index in our participants, we conducted Pearson’s correlations between CogWM Improvement Index and number of sessions completed, as well as baseline CDRS-R, YMRS, CPRS-R, ADHD Total Index scores, and BRIEF WM scores. A Bonferroni correction was applied on the standard threshold for significance (*P* < 0.05), with a resulting corrected *P* = 0.008. The results revealed no significant correlation with number of sessions completed (*P* = 0.81), baseline CDRS-R scores (*P* = 0.48), baseline YMRS scores (*P* = 0.51), baseline CPRS-R ADHD total index scores (*P* = 0.61), or baseline BRIEF-PR WM scores (*P* = 0.75).

## Discussion

To our knowledge, this study is the first to examine the effects of WM training concurrently in youths with ADHD and youths with PBD. Furthermore, our findings suggest that WM training is beneficial not only in youths with ADHD but also in youths with PBD, with or without ADHD comorbidity.

Our first hypothesis was confirmed, in that both the PBD and the ADHD group significantly improved their performance on the Cogmed tasks after training, as measured by the CogWM Improvement Index. The two groups did not differ significantly on Cogmed WM performance at baseline or on the Cogmed WM Improvement Index after training. The current findings provide important additional evidence of WM improvement in children with ADHD after cognitive training (Klingberg et al., 2004; Holmes et al., 2009; Thorell et al., 2009). Importantly, a novel finding is that the cognitive intervention was beneficial also to children suffering from severe mood dysregulation, i.e., youths with a primary diagnosis of PBD.

Because about half of the PBD participants had ADHD comorbidity, we conducted covariate analyses to study any potential effects of ADHD comorbidity on the results. Our findings indicate that for the vast majority of our measures, ADHD comorbidity did not significantly affect PBD performance before or after training. There were, however, a few notable exceptions as follows. Regarding TMT B, we found a significant effect of ADHD comorbidity on PBD performance, in that before the training the PBD group had higher (i.e., worse) scores than the Comorbid group and the ADHD group. Moreover, there was a significant effect of ADHD comorbidity on PBD post-training CPRS-R scores, for the Hyperactivity scale. These data indicate that for hyperactivity symptoms, PBD patients with ADHD comorbidity benefited more from the training than patients with PBD only. Finally, regarding the BRIEF-PR, we found a significant effect of ADHD comorbidity on PBD scores only for the WM scale at baseline, such that the Comorbid group had worse WM deficits than the PBD group. However, after the training, the effect of comorbidity was not significant anymore for this measure. Taken together, these findings suggest that ADHD comorbidity effects were related mostly to baseline performance and tended to disappear after the training, in that for certain domains, the Comorbid group seemed to benefit more from the cognitive training than the PBD-only group.

While in the past WM capacity was seen as a non-malleable trait, our current results, together with recent developmental studies (Klingberg et al., 2005; Westerberg and Klingberg, 2007; Holmes et al., 2009; Klingberg, 2010; Stevens et al., 2015; Lengvenyte et al., 2019), suggest that WM can indeed be improved in youths with WM deficit, including PBD youth, through a relatively short and home-based computerized cognitive training.

Generally, our results are in line with findings of improved verbal and spatial WM performance in the one published study on WM intervention in adults with BD (Lengvenyte et al., 2019). However, a more direct comparison of results is not possible because of the different clinical and neuropsychological tests adopted in the two studies. Presently, there is still a much more extensive literature on the effects of cognitive intervention in adult patients with schizophrenia than in adult patients with BD. Dickinson et al. (Haldane et al., 2008; Dickinson et al., 2010) and Murthy et al. (2012) found improvements in training exercises in individuals with schizophrenia, although there was no generalization of the improvement to non-trained cognitive tasks. However, there is also evidence of “far transfer” in individuals with schizophrenia, with findings of improved neuropsychological performance (Fisher et al., 2009) and psychosocial functioning (Mcclure et al., 2005; Drapier et al., 2008) after cognitive remediation.

Our study collected only behavioral data, and therefore, we cannot speak directly to any effects of cognitive training on brain function. However, there are some recent publications that speak to the issue of the neurological effects of WM training. For instance, Stevens et al. (2015) reported that after Cogmed training, adolescents with ADHD exhibited improvements not only in ADHD symptoms and WM performance but also in brain activity in frontal, temporal and parietal regions (i.e., WM circuits). Neural changes in prefrontal and parietal regions and changes in density of dopamine D1 receptors were also previously found in healthy adults who underwent Cogmed training (Olesen et al., 2004; Tillman et al., 2008). Moreover, a cognitive remediation study with individuals with schizophrenia showed that improvements in attention and reality monitoring were accompanied by increased activity in medial prefrontal cortex (Subramaniam et al., 2012). While more scientific evidence is needed, these brain imaging findings suggest that training-related cognitive improvements may be mediated by fairly specific changes in the fronto-cingulate-parietal circuit. This circuit plays a key role in WM functions (D’esposito et al., 2000; D’esposito, 2007) and is markedly impaired in PBD (Pavuluri and Passarotti, 2008; Passarotti et al., 2010a, b; Passarotti and Pavuluri, 2011). Therefore, a goal of future studies should be to systematically examine whether behavioral performance improvements in ADHD and PBD youths may be related to significant changes in WM circuits.

A second important study goal was to examine whether there is generalization of WM improvement to “non-trained” neuropsychological tasks that rely on similar cognitive skills as the trained tasks, which would exemplify “near transfer.” Our hypothesis for “near transfer” was confirmed by the results, although not always in both groups. Specifically, after training, only the PBD group showed a significant improvement in performance for TMT A, a task requiring basic attention and WM processes. Furthermore, after training, both the PBD and ADHD groups exhibited significant improvement on the Digit Span Test, a task that relies on verbal WM. We also found that for the Digit Span Test, PBD improved significantly on the LDSB score, a measure of backward trial performance, while ADHD improved significantly on the LDSF score, a measure of forward trial performance. Only ADHD exhibited significant improvement on the Spatial Span Test, engaging visuospatial WM processes. Moreover, on this test ADHD showed an improvement only on LDSF but not on LDSB trials. In sum, our results on the TMT A, Digit Span, and Spatial Span tasks provide evidence of “near transfer” of WM improvement to verbal and visuospatial attention functions after Cogmed training, in line with other published developmental studies (Kronenberger et al., 2011; Spencer-Smith and Klingberg, 2015; Stevens et al., 2015; Van Der Donk et al., 2015; Randall and Tyldesley, 2016).

Our third study goal was to examine potential generalization of WM improvement to non-trained tasks, which would provide evidence of “far transfer.” In this regard, we obtained some significant results, either from the PBD group or from the ADHD group. The first piece of evidence of “far transfer” was found in the PBD group, who exhibited significant improvements on several subscales of the BRIEF-PR scale. Before training, PBD had clinically elevated scores on all the BRIEF subscales, but after training, this group improved on Inhibition, as well as the BRI and GEC indexes. Importantly, both indexes include the Emotional Control subscale, which is clinically relevant because of the extensive mood dysregulation present in PBD. Note that while parents who are not blind to treatment may be biased to report improvement after intervention, only specific improvements on specific BRIEF-PR subscales were found here, which suggests specificity of improvement. Furthermore, before training, PBD had more severe scores than ADHD on several BRIEF subscales, such as Inhibition, Shifting, Emotional Control, Monitoring, as well as the BRI and GEC. However, after training, the only subscale where PBD had still significantly worse scores than ADHD was the Emotional Control scale. This finding is to be expected given that PBD usually presents with much more severe mood dysregulation than ADHD. This pattern of results suggests a greater improvement in executive functions in PBD that partially normalized its scores reaching the ADHD group levels. It is noteworthy that we were able to find a significant improvement in executive function domains that are particularly challenging in PBD, such as inhibition and behavioral regulation, after a training that is focused on basic WM functions, rather than more complex self-regulation skills. These results are in line with other findings of “far transfer” in children after WM training (Klingberg et al., 2005; Holmes et al., 2009, Stevens et al., 2015). The ADHD group did not show any significant improvement on the BRIEF-PR scale, even though the baseline scores improved slightly after training. This is possibly due to the fact that ADHD, unlike PBD, was already on a regimen of ADHD medications that adjusted cognitive symptoms, leading to milder deficits than PBD on the BRIEF-PR assessment even at baseline.

The second and third piece of evidence of “far transfer” are related to the ADHD group. Specifically, the ADHD group showed an improvement in the TMT B after training, suggesting an improvement in cognitive flexibility and executive functions, which is considered “far transfer” because these domains that were not directly targeted by the WM training. Moreover, the ADHD group showed “far transfer” in terms of a significant improvement on the Reading Fluency Test of the Woodcock–Johnson III, a test where at baseline this group had scored below average. This result is in line with studies showing a very close relation between WM and reading (Gathercole et al., 2016), such that improved WM functions lead to improved reading skills. Conversely, the PBD group did not show any improvement on this test, probably because it exhibited already average scores at baseline, which may have limited the extent of any potential improvement. No significant results in either group were obtained for the Math Fluency Test, possibly because the Math Fluency test involves more specific abstract reasoning skills that were not much engaged by the Cogmed training.

Our results do not suggest any evidence of “far transfer” with regard to ADHD symptoms or inhibition functions. Unlike the findings by Stevens et al. (2015) with ADHD youths, our findings did not show any significant improvement in ADHD symptoms, as measured by the CPRS-R, in either group after the training. There were also no significant improvements in either group on the SST. The SST poses high demands concurrently on WM, attention, inhibition, and EF in the presence of prepotent motor responses, and therefore, it may benefit from a more specific “inhibition-focused” training, rather than a WM training like the one adopted here.

The fourth piece of evidence of “far transfer” was with regard to mood, and specifically depression symptoms in ADHD. The ADHD group showed a significant reduction in CDRS-R scores after the training. While we do not have a clear explanation for this finding, we could speculate that the cognitive training may indirectly improve the child’s sense of competence, as well as the quality of child–parent interactions. This may result in improved self-confidence, and a reduction, even if maybe temporary, in depressive symptoms in children with ADHD. While replications are needed, our initial findings suggest a tangible possibility that cognitive training may benefit patients presenting with depression. Interestingly, a meta-analysis on computerized cognitive training in patients with major depressive disorder also found improvements in symptoms of depression, together with improvements in attention, WM, and global functioning (Motter et al., 2016).

With regard to the key question of whether the benefits of training would persist in time, preliminary data from a very modest subsample of patients indicated that, after 4 months, the patients were still retaining the training benefits on WM performance, with no significant differences between performance scores at the end of training and scores after 4 months. This was true for tasks where the patients had shown significant improvements after training (TMT A and B, Digit and Spatial Span Test, BRIEF-PR) as well as for other tasks that had shown no improvements. It is noteworthy that, for the Digit Span Task (which is very similar to several of the trained Cogmed WM tasks), we actually found a significant improvement in scores after 4 months compared to the end of training. Interestingly, a study by Kronenberger et al. (2011) in children with cochlear implants found that post-training WM improvements on various tests decreased slightly at 1-month follow-up and more significantly at 6-month follow-up. However, a sentence repetition task that was part of their cognitive battery showed a significant improvement even at 6-month follow-up. While the present preliminary findings need to be considered with much caution because of the very small sample, they nonetheless suggest a potential for retention of the training benefits in time, possibly more so for some cognitive domains as compared to others. If these results were to be replicated, they would increases the functional and clinical usefulness of cognitive intervention in youths with BD and ADHD. It is still an open question whether the noted improvement may be retained for a longer period than 4 months after training and also whether additional “booster” sessions may benefit retention of the improvement over longer periods of time.

With regard to identifying predictors for the WM improvement in our participants, exploratory correlation analyses revealed no significant correlations with number of sessions completed, baseline CDRS-R, YMRS, CPRS-R, or BRIEF-PR WM scores, suggesting that, at least in our samples, there is not a strong relationship between WM improvement after training and severity of ADHD, mood, or WM symptoms at baseline. Future studies with larger samples will be needed to better explore predictors of WM improvement in pediatric patients.

It is noteworthy that the contributions of cognitive training in patient population may have the potential to go beyond cognitive improvement *per se*. Working memory and executive functions have been linked to functional outcome, and therefore, improving deficits in these domains may benefit functional outcome. A study by Lantrip et al. (2015) found that in 12- to 18-year-old adolescents, greater ability to use reappraisal as an emotion regulation strategy was associated with better executive functions, possibly because they enable an individual to use more cognitive resources and strategies to cope more efficiently with life challenges. Therefore, by strengthening fronto-cingulate-parietal circuits involved in executive functions through WM training, we may be able to improve not only WM but also cognitive control and self-regulation in PBD and ADHD. To reach long-lasting effects, the cognitive training would need to be part of a multilevel clinical intervention including motivational interviewing (Mohr et al., 2013), mood stabilization in PBD, symptoms monitoring, and cognitive–behavioral therapy intervention, in PBD and ADHD (West and Pavuluri, 2009).

### Study Limitations

Our findings should be considered in light of several limitations.

The children with PBD were recruited from the University Clinic, while the children with ADHD were recruited both from the University Clinic and the community. This may have resulted in a more functionally impaired PBD group relative to the ADHD group. The study samples are relatively small. Larger samples from different recruitment sources may increase statistical power. Moreover, in this study, there was not an HC group, and the patient groups were their own control in terms of cognitive training effects. Future studies will need to include a HC group to test whether patient post-training performance may normalize to HC levels or not. Future replications will also need to follow a double-blind randomized controlled trial design.

The vast majority of our patients were medicated. For both ethical and practical reasons, we decided not to ask patients to get off their medications for this study. In addition, since the vast majority of our patients were medicated, we could not covary medication in analyses, and therefore, we cannot generalize our results to non-medicated PBD and ADHD youths. However, given the age range considered, it would be very difficult to find patients with a diagnosis of PBD or ADHD who are not professionally monitored and on medications. Furthermore, our experience suggests that it may be very difficult for unmedicated patients to be able to focus and engage in the cognitive training while dealing with significant attentional deficits or emotional symptoms. Note that we do not propose Cogmed as an alternative to pharmacological treatments. Rather, we suggest that the cognitive training may be more beneficial when combined with a medication regimen in psychiatric patients.

Our results on 4-month follow-up data need to be interpreted with caution because of the very small sample. Future studies need to improve participant retention and further investigate the degree to which any training-related improvement may persist in the months following the end of training.

Our testing battery included only one computerized cognitive performance task, the SST, to measure response inhibition. Future studies will benefit from multiple computerized tasks examining additional cognitive domains such as verbal and visuospatial WM, selective and sustained attention, and EF to provide converging evidence to the neuropsychological test results.

## Conclusion

In sum, our results provide preliminary evidence that a computerized training program can improve WM function and support “near” and “far” transfer in youths with PBD and youths with ADHD, although in different ways for the two patient groups. Future studies examining the “mechanisms” of cognitive enhancement in different pediatric populations with mood dysregulation or ADHD will ultimately aid in tailoring more effective, illness-specific cognitive interventions.

## Data Availability Statement

The datasets generated for this study are available on request to the corresponding author.

## Ethics Statement

The studies involving human participants were reviewed and approved by IRB Committee, The University of Illinois at Chicago. Written informed consent to participate in this study was provided by the participants’ legal guardian/next of kin.

## Author Contributions

AP was the study Principal Investigator. She designed the study, supervised testing, met with patients and families, trained personnel, supervised the cognitive training, carried out some of the analyses, and wrote the manuscript. LB and NT conducted some testing, organized and stored data, analyzed some data, read and edited the manuscript. LC conducted some testing, read and edited the manuscript. LK analyzed some data, read and edited the manuscript. LL provided statistical consulting, read and provided comments on the manuscript. SL advised on parts of the data analyses, read and edited the manuscript.

## Conflict of Interest

The authors declare that the research was conducted in the absence of any commercial or financial relationships that could be construed as a potential conflict of interest.
