# Supplementary material for: A Preliminary Study on the Functional Benefits of Computerized Working Memory Training in Children With Pediatric Bipolar Disorder and Attention Deficit Hyperactivity Disorder
Source: Front Psychol. 2020 Feb 5;10:3060. doi: 10.3389/fpsyg.2019.03060 (PMC7014966; doi:10.3389/fpsyg.2019.03060)
Supplement: Supplementary file 1 [file Table_1.pdf]

Supplementary Material

Supplementary Table 1

Cogmed Tasks Description.

Task	Description
Input Module	Numbers are read aloud and shown on the screen. The objective is to remember the sequence and reproduce them in the reverse order.
Input Module with Lid	This is a slightly different version of the Input module exercise. The numbers are read aloud, however the number buttons are covered by a lid.
Decoder	Certain letters will be read out loud. The objective is to recall and reproduce them in the correct order.
Visual Data Link	A number of lights come on in succession. The objective is to remember the order and click on the lights in the same order.
Rotating Dots	A dial (much like a rotary phone) with many lights will be turning and lights will light up as it rotates. The objective is to remember the sequence as it turns and then click on the correct lights even as it continues to turn.
Stabilizer	Certain letters will be read aloud, and a light will come on in a circle display. The objective is to remember which letter corresponds to which light. When one of the letters is displayed in the circle the participant will need to click on the correct light previously associated with that letter.

Data room	The inside of a 3D room is presented with each side being comprised of four lights which will randomly light up. The objective is to remember the order in which the lights light up and repeat them back by clicking on the appropriate lights.
Rotating Data Link	Lights come up in a varying sequence and then the entire panel turns 90 degrees. The objective is to remember the sequence even after the panel has rotated by clicking on the appropriate lights.
Sorter	Certain numbers behind latches will be shown one at a time. The objective is to click and sort them in succession (e.g., 1-2-3).
3D Cube	A 3D cube will be presented with each side being comprised of four square panels which will randomly light up. The objective is to remember the order in which the square panels light up and repeat them back by clicking on the appropriate lights.
Space Whack	A cloud of mist comes out of various craters in a random order. After the mists have gone off a monster will appear in each crater. Once the cloud is gone and the monster appears, the participant is to click on the monsters in the remembered order.
Asteroids	There will be asteroids moving about randomly on the screen, and will light up in a certain order. The participant is to remember the order in which the asteroids light up even as they continue to move about the screen and click on the asteroids in the same order they lit up.
